# Antigen Production After Latency Reversal and Expression of Inhibitory Receptors in CD8+ T Cells Limit the Killing of HIV-1 Reactivated Cells

**DOI:** 10.3389/fimmu.2018.03162

**Published:** 2019-01-22

**Authors:** Alba Ruiz, Oscar Blanch-Lombarte, Esther Jimenez-Moyano, Dan Ouchi, Beatriz Mothe, Ruth Peña, Cristina Galvez, Meritxell Genescà, Javier Martinez-Picado, Philip Goulder, Richard Barnard, Bonnie Howell, Bonaventura Clotet, Julia G. Prado

**Affiliations:** ^1^IrsiCaixa AIDS Research Institute, Badalona, Spain; ^2^Germans Trias i Pujol Research Institute (IGTP), Universitat Autonoma de Barcelona, Badalona, Spain; ^3^Faculty of Medicine, University of Vic - Central University of Catalonia (UVic-UCC), Vic, Spain; ^4^Department of Infectious Diseases, Hospital Universitari Vall d'Hebrón, Institut de Recerca (VHIR), Universitat Autònoma de Barcelona, Barcelona, Spain; ^5^Catalan Institution for Research and Advanced Studies (ICREA), Barcelona, Spain; ^6^Department of Paediatrics, University of Oxford, Oxford, United Kingdom; ^7^Department of Infectious Disease, Merck & Co. Inc. Kenilworth, NJ, United States

**Keywords:** human immunodeficiency virus, HIV-1 reservoir, HIV-1 immunogen, shock and kill, CTL (Cytotoxic T lymphocyte), inhibitory receptors

## Abstract

The so-called shock and kill therapies aim to combine HIV-1 reactivation by latency-reversing agents (LRA) with immune clearance to purge the HIV-1 reservoir. The clinical use of LRA has demonstrated detectable perturbations in the HIV-1 reservoir without measurable reductions to date. Consequently, fundamental questions concerning the limitations of the recognition and killing of LRA-reactivated cells by effector cells such as CD8+ T cells remain to be answered. Here, we developed a novel experimental framework where we combine the use of cytotoxic CD8+ T-cell lines and *ex vivo* CD8+ T cells from HIV-1-infected individuals with functional assays of LRA-inducible reactivation to delineate immune barriers to clear the reservoir. Our results demonstrate the potential for early recognition and killing of reactivated cells by CD8+ T cells. However, the potency of LRAs when crossing the barrier for antigen presentation in target cells, together with the lack of expression of inhibitory receptors in CD8+ T cells, are critical events to maximize the speed of recognition and the magnitude of the killing of LRA-inducible provirus. Taken together, our findings highlight direct limitations in LRA potency and CD8+ T cell functional status to succeed in the cure of HIV-1 infection.

## Introduction

The introduction of antiretroviral treatment (ART) has increased life expectancy and improved the health of people living with HIV-1 infection. However, ART does not cure HIV-1 infection, and treatment is needed for decades, thus raising the alarm about long-term health and sustainability of treatment and care of HIV-1-infected individuals (1). The current limitations of ART highlight the need for therapeutic strategies to eradicate HIV-1 from the body. A major obstacle to eradication is the establishment of the HIV-1 reservoir (2). Besides, the reservoir is perpetuated through cellular homeostatic proliferation and clonal expansion, even after years of effective ART (3, 4).

One therapeutic approach, the so-called shock and kill strategies, has been tested in several clinical trials and proposes the use of latency-reversing agents (LRA) for the transcriptional activation of HIV-1 (shock) and the clearance of reactivated cells by immune responses (kill). Although several studies have demonstrated reactivation of HIV-1 by LRA both *in vitro* and *in vivo* (5–7), no measurable reduction in the HIV-1 reservoir has been found to date (8). Consequently, ensuring the immune recognition of LRA-reactivated cells by effector responses will be essential for eradication of the HIV-1 reservoir (9, 10). Several studies have proposed CD8+ T cells as effector cells for recognition and clearance of LRA-reactivated cells (11) based on their ability to control the reservoir size in natural controllers (12–14), their potent *in vitro* antiviral activity (15, 16), and their role in controlling viral replication despite ART (17). Although the frequency of HIV-1–specific CD8+ T cells decays with ART (18, 19), the cells retain effector and cytotoxic properties that enable them to recognize and kill HIV-1-infected cells (11, 17, 20, 21). However, functional barriers to CD8+ T-cell antiviral activity upon treatment with LRA can affect the success of shock and kill strategies. These barriers may be associated with CD8+ T-cell dysfunction, which is a consequence of LRA treatment itself, and with the pro-inflammatory environment driven by HIV-1 infection. Several studies suggest an immunosuppressive effect of LRA, particularly histone deacetylase inhibitors (HDACi), on CD8+ T-cell antiviral activity (22, 23). Data remain controversial, and while some studies suggest a time-dependent or direct effect of HDACi on CD8+ T-cell function (24), others do not find a measurable impact on *ex vivo* CD8+ T-cell function after *in vivo* administration of HDACi (7). Moreover, the chronic pro-inflammatory environment and the persistence of antigen exposure affect the functional profile of HIV-1–specific CD8+ T-cell responses (25, 26). This pro-inflammatory environment leads to the reduction of cytotoxic potential and the upregulation of inhibitory receptors, such as PD-1, LAG-3, and TIM-3 in CD8+ T cells associated with dysfunction and immune exhaustion in HIV-1-infected individuals (27–30).

In this context, fundamental questions regarding the limitations of LRA activity on target cells and CD8+ T-cell sensing in response to HIV-1 reactivation remain unanswered. In this study, we design a novel experimental framework where we combine cytotoxic HIV-1 CD8+ T-cell lines (CTL) and *ex vivo* CD8+ T cells from HIV-1-infected individuals with an *in vitro* model of LRA-dependent HIV-1 reactivation. In this framework, we evaluate the so-called window of opportunity between latency reversal and killing of reactivated cells by CD8+ T cells. We characterize HIV-1 protein expression upon treatment with LRA and its association with antigen presentation and delineate the kinetics of recognition and killing of HIV-1 reactivated cells by CD8+ T cells. We also analyze the functional limitations of CD8+ T cells from HIV-1-infected individuals in the elimination of reactivated cells. We observed a correlation between LRA potency and the speed and magnitude of the killing of reactivated cells by CD8+ T cells. Although we found increased killing of reactivated cells by *ex vivo* CD8+ T cells in response to LRA, the magnitude of the response was highly variable across HIV-1-infected individuals and was associated with a lack of expression of inhibitory receptors in CD8+ T cells. Our data highlight several limitations in the efficacy of shock and kill strategies and point to the need for a trade-off between LRA potency and CD8+ T-cell functional status in HIV-1-infected individuals if the reservoir is to be cleared.

## Results

### LRA Allow HIV-1 Protein Expression and HLA-Class I Antigen Presentation for CD8+ T-Cell Recognition to Increase Killing of Latently Infected Cells

**Figure 1:**
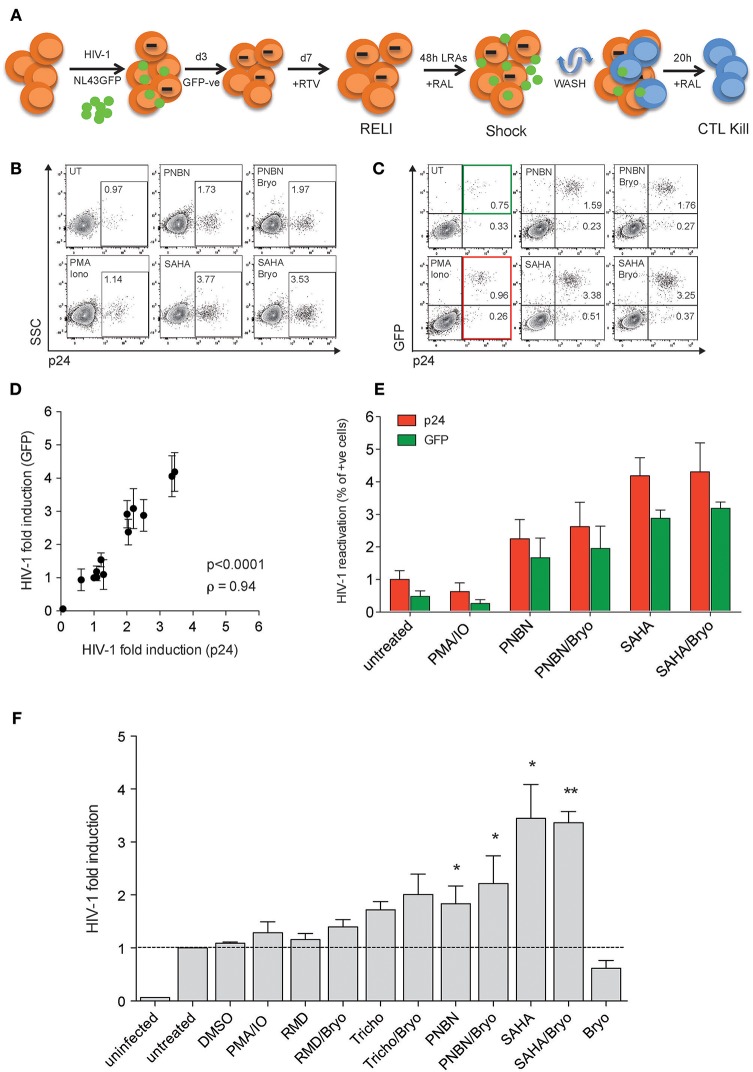
The HIV-1 RELI “shock and kill”model. **(A)** Schematic representation of the HIV-1 RELI model for “shock and kill.” RELI cells were reactivated with LRA in the presence of RAL before being extensively washed and cultured in the presence or absence of HLA-class I–matched CTL. After 20 h of co-culture, we evaluatedHIV-1 reactivation (shock) and the killing of HIV-reactivated by CTL (kill). **(B)** Representative dot plots of intracellular p24 expression in live RELI cells under untreated conditions (UT) compared with PMA/Ionomycin and SAHA and PNBN alone or combined with Bryo. **(C)** Representative dot plots of intracellular expression of p24 and GFP from live RELI cells reactivated with LRA. The green square indicates GPF- positive cells, and the red square indicates p24-positive cells. **(D)** Correlation between HIV-1 fold induction measured according to the expression of p24 or GFP in RELI cells after LRA treatment. The HIV-1 fold induction was calculated as the ratio between % of p24- or GFP-expressing cells under LRA conditions and the % of p24- or GFP- expressing cells left untreated. The line indicates the fit of the data to a linear regression. The Spearman correlation coefficient (ρ) and the two-tailed *p-*value are shown. The graph represents the mean ± SEM of three independent experiments performed in replicates. **(E)** HIV-1 reactivation measured by the percentage of p24 positive cells or GFP-positive cells. The graph represents the mean ± SEM of three independent experiments performed in replicates. **(F)** HIV-1 fold induction of RELI cells. The HIV-1-fold induction was calculated as the ratio between the % of p24 expressing cells under LRA conditions and the % of p24-expressing cells in the left untreated. The data represents the mean ± SEM of five independent experiments performed in replicates. The *p*–values were calculated using the one-sample *t-*test. Only significant values are shown in the figure (^*^p < 0.05, ^**^p < 0.005).

First, we developed the “resting-like” or RELI-model to evaluate HIV-1 reactivation by LRA (shock) simultaneously with the elimination of reactivated cells by HIV-1-specific cytotoxic CD8+ T-cell lines (CTL) (kill), as schematized in Figure 1A and detailed in the Materials and Methods section. Briefly, we infected U937-HLA-B^*^27:05 cells with the HIV-1_NL43GFP_ reporter virus as previously reported (31, 32). After 72 h of infection, we sorted the GFP negative cells and cultured them for 4 days in the presence of the protease inhibitor ritonavir (RTV). At this point, we obtained a RELI population enriched in uninfected and non-productively HIV-1-infected cells. RELI cells were then washed, treated with the HIV-1 integrase inhibitor raltegravir (RAL) to avoid multiple rounds of infection, and exposed to LRA shock for 48 h. We also treated RELI cells with azidothymidine (AZT) to avoid the formation of unintegrated episomal forms that may contribute to the expression of viral proteins (data not shown) (33). LRA-treated cells were then extensively washed to uncouple the LRA effect from CD8+ T-cell function against reactivated cells (24), and co-cultured with HLA-class I–matched HIV-1-specific CTLs for 20 h (kill) (Figure 1A).

We measured HIV-1 reactivation in RELI cells in response to LRA for 48 h including the HDACi panobinostat (PNBN), romidepsin (RMD), trichostatin A (Tricho), and suberoylanilide hydroxamic acid (SAHA). We also included combinations of HDACi and bryostatin A (Bryo) to evaluate potential additive effects between HDACi and protein kinase C agonists (PKCag) as previously reported (34). Drug concentrations were selected based on previous clinical or *in vitro* studies (7, 35–37) and in the absence of direct cellular toxicity (Table S1). The use of HIV-1_NL43GFP_ enabled us to monitor viral reactivation by flow cytometry using two markers the expression of p24 protein (Figure 1B) and the expression of GFP encoded in frame with the HIV-1 Nef protein, as previously reported (38, 39) (Figure 1C). We observed a direct correlation between HIV-1 fold induction measured according to p24 or GFP expression (*p* < 0.0001; ρ = 0.94, Figure 1D). In this context, the expression of p24 was consistently higher than that of GFP (p24 vs. GFP *p* < 0.005, Wilcoxon matched-pairs signed-rank test) (Figure 1E), thus indicating its greater sensitivity as a marker for viral antigen expression upon reactivation and supporting its application in subsequent experiments. However, the use of GFP expression as a marker of HIV-1 induction is particularly relevant given the correlation between p24 and GFP expression for the screening of large compound libraries and, thus, the increased cost-effectiveness of the method.

As shown in Figure 1F, we compared HIV-1 reactivation after LRA treatment with Tricho, PNBN, and SAHA alone or combined with Bryo with untreated or DMSO controls. The levels of HIV-1 reactivation ranged between a 2- and 4-fold viral induction, with a significant increase for PNBN and SAHA alone (*p* < 0.05) or combined with Bryo (*p* < 0.005). The additive effects of combining HDACi/Bryo were modest or absent.

**Figure 2:**
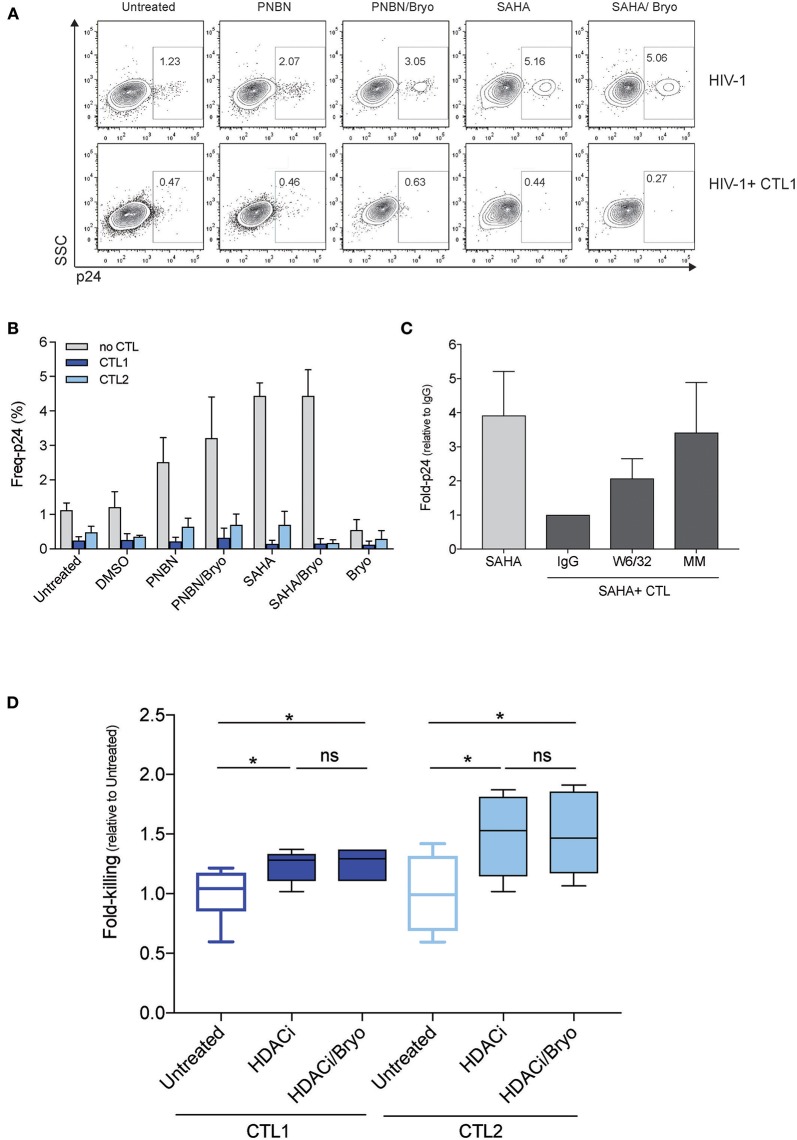
LRA treatment increased the magnitude of killing of HIV-1 reactivated cells by CD8+ T cells. **(A)** Representative dot plots showing the frequency of p24 cells from live HIV-1 reactivated cells in the absence (top) or in the presence of CTL1 for 20 h. **(B)** Frequency of p24 cells from HIV-1 reactivated cells inthe absence or presence of CTLs (CTL1 and CTL2). The graph represents the mean ± SEM of three independent experiments performed in duplicate. **(C)** Fold-p24 expression relative to SAHA-reactivated cells in the presence of HLA-matched CTLs treated with isotype control antibody IgG. SAHA-reactivated cells in the absence or presence of HLA-matched CTLs (CTL1, CTL2), treated isotype control antibody IgG (IgG), and HLA-blocking antibody (W6/32), and in the presence of HLA-mismatched CTL3 (MM). The graph represents the mean ± SD of three experiments performed in duplicate. **(D)** Relative killing of RELI cells by untreated CTL1 and CTL2 and presence of HDACi (including PNBN and SAHA) alone and combined with Bryo. Box and whisker plots included data from four independent experiments performed in duplicate. The *p*-values were calculated using the Mann-Whitney test. Only significant values are shown in the figure (^*^p < 0.05).

Next, we assessed whether LRA treatment enabled HIV-1 antigen presentation in the context of HLA-class I molecules for their recognition and killing by HIV-1 cytotoxic CD8+ T-cell lines (CTL). For this purpose, we used two CTLs, CTL1, and CTL2, which recognized HLA-B^*^27:05 restricted epitopes (Gag KK10 and Pol KY9, respectively) as previously described (31). Briefly, we exposed RELI cells to PNBN and SAHA alone or combined with Bryo and co-cultured them with CTLs restricted by HLA-B^*^27:05. After 20 h of co-culture, we assessed killing by measuring the frequency of p24-expressing cells in the absence or presence of CTL1 (Figure 2A and Figure S1). As shown in Figure 2B, we observed significantly increased killing by CTL1 and CTL2 after LRA treatment (*p* < 0.0005, one-way ANOVA). In fact, CTL1 and CTL2 were highly functional and had similar 50% effective concentrations (EC_50_, −4.8, and −5.5, respectively), despite differences in epitope specificity and a higher response for CTL1 at low HIV-1 antigen concentrations (Figure S2).

In addition, we added an anti-HLA-A/B/C (W6/32) antibody or an HLA-mismatched (MM) CTL3 to demonstrate that, upon viral reactivation, CTL killing was mediated through contacts with class I HLA HIV-1 antigen complexes. The elimination of reactivated cells was significantly abolished both by means of the W6/32 antibody and by impeding the recognition of the HLA-class I peptide complexes with an HLA-MM co-culture (*p* < 0.05, one-way ANOVA) (Figure 2C). Thus, the compiled analysis of all LRA tested showed a significant increase in the magnitude of killing of cells latently infected by HIV-1 after LRA treatment in both CTL (*p* < 0.05, Figure 2D). However, we did not observe any additional benefit in the frequency of reactivated cell killing through the combination of HDACi with Bryo (Figure 2D).

These data confirm that inducible reactivation of HIV-1 by LRA enables antigen presentation in the context of HLA-class I molecules to increase killing of latently infected cells by CTL.

### LRA Potency Modulates the Speed of HIV-1 Antigen Recognition and the Magnitude of the Killing of Reactivated Cells by CD8+ T Cells

**Figure 3:**
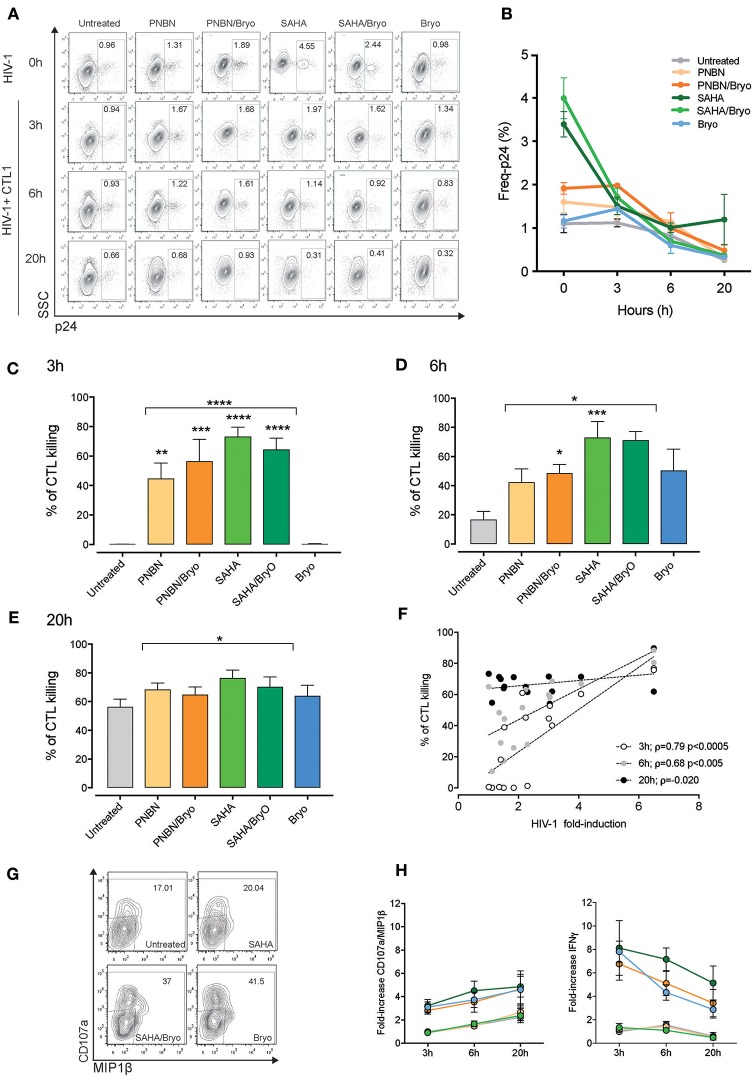
Kinetics of killing in response to HIV-1 reactivation by CTL. **(A)** Representative dot plots showing the percentages of intracellular p24 from HIV-1 RELI cells. The top line indicate the level of HIV-1 reactivation of RELI cells at baseline (0 h) in untreated and LRA-treated conditions and 3, 6, and 20 h after co-culture with CTL1 **(B)** Kinetics of elimination of HIV-1 reactivated by CTL. The graph shows the mean ± SEM of two independent experiments for CTL1. **(C)** Kinetics of killing of HIV-1 reactivated cells by CTLs at 3 h; **(D)** at 6 h; **(E)**, and at 20 h after co-culture. Values in graphs C to E correspond to the mean ± SEM of three independent experiments for CTL1 and three independent experiments for CTL2 in replicates. The *p*-values were calculated using the unpaired *t*-test for comparisons between untreated cells and cells treated with HDACi (top line) and one-way ANOVA for multiple comparisons (asterisks above the bars). Only significant *p*-values are represented in the figure (^*^p < 0.05, ^**^p < 0.005, ^***^p < 0.0005, ^****^p < 0.00005). **(F)** Correlation between HIV-1 fold induction and frequency of killing mediated by CTLs. The line indicates the fit of the data to a linear regression. The Spearman correlation coefficient (ρ) and the two-tailed *p*-value are indicated. The graph represents the frequency of killing from three independent experiments for CTL1 in replicates and three independent experiments for CTL2 in replicates. **(G)** Representative dot plots showing percentages of CD107a/MIP1β from CTL1 after co-culture with HIV-1 reactivated RELI cells. **(H)** Kinetics of CD107a/MIP1β and IFNγ secretion from CTLs after co-culture with HIV-1 reactivated RELI cells. The graphs show the mean ± SEM of two independent experiments for CTL1 and two independent experiments for CTL2 in replicates.

It is essential to investigate the kinetics of recognition and killing of LRA-reactivated cells and to establish a window of opportunity between shock and kill by CD8+ T cells for therapeutic optimization. By using the RELI model, we monitored the kinetics of recognition and elimination of HIV-1 reactivated cells by CTLs at 3, 6, and 20 h post co-culture. We measured changes in the frequency of p24-positive cells from time 0 by flow cytometry at 3, 6, and 20 h after co-culture of untreated and LRA-treated RELI cells with CTLs (Figure 3A). We observed a reduction in the frequency of p24-expressing cells over time in culture with CTLs (Figure 3B). HIV-1 reactivated cells were eliminated rapidly after 3 h of co-culture in SAHA, PNBN, SAHA/Bryo, and PNBN/Bryo conditions compared with untreated cells for both CTLs (untreated vs. HDAC ± Bryo; *p* < 0.0001) (Figure 3C). These differences were sustained at 6 h after co-culture (untreated vs. HDAC ± Bryo; *p* < 0.0005) and 20 h after co-culture (untreated vs. HDAC ± Bryo; *p* < 0.005) (Figures 3D,E). These data indicate a general increase in the speed of recognition and killing of reactivated cells by CTLs starting at 3 h in the presence of PNBN, PNBN/Bryo, SAHA, and SAHA/Bryo, although our results do not reveal a hierarchy for LRAs. The data suggest a prolonged effect over the first 6 h by PNBN/Bryo, SAHA, and SAHA/Bryo compared with untreated cells (Figures 3D,E) (untreated vs. PNBN/Bryo; *p* < 0.05, untreated vs. SAHA, *p* < 0.005; and untreated vs. SAHA/Bryo; *p* < 0.005). By contrast, the use of Bryo alone did not significantly increase the speed or magnitude of killing compared with untreated condition at 3, 6, or 20 h of co-culture (Figures 3C–E). Although we observed a marginal augment in the killing at early time points, suggesting an indirect effect of Bryo in CTL activation despite extensive washout of the drug as previously reported (24).

Interestingly, as shown in Figure 3F, we found a direct correlation between the magnitude of CTL killing and the levels of HIV-1 inducible reactivation by LRA at early time points (3 h *p* < 0.0005, ρ = 0.74; 6 h *p* < 0.005, ρ = 0.68), thus indicating that the potency of the LRA for HIV-1 antigen expression dictates the speed of CTL recognition. Based on these data, we propose a threshold of >2-fold HIV-1 induction upon LRA treatment to allow antigen presentation and engage rapid recognition and killing of HIV-1 reactivated cells by CTL.

Besides, we investigated whether HIV-1 killing by CTL can be monitored by changes in cytokine expression and degranulation upon antigen recognition using CTL as biosensors (40), we measured CD107a/MIP1β and IFNγ expression (Figure 3G, dot plots). Despite the increase of HIV-1 expression by LRAs, the use of SAHA or PNBN did not alter the kinetics of CD107a/MIP1β or IFNγ expression in the tested CTLs, as compared with untreated conditions. By contrast, Bryo indirect effects increased both CD107a/MIP1β and IFNγ secretion by CTLs at early time points (Figure 3H). We also found different profiles for CD107a/MIP1β and IFNγ expression over time, with increasing frequencies for CD107a/MIP1β and decreasing frequencies for IFNγ secretion. Thus, the kinetics of cytokine expression by CTLs did not mirror changes associated with HIV-1 inducible reactivation or killing.

Overall, these data indicate a direct relationship between the potency of inducible reactivation of HIV-1 by LRA and the speed of recognition and killing of reactivated cells by CD8+ T cells. Therefore, the potency of LRA to cross the threshold of HIV-1 antigen presentation in reactivated cells is a crucial event to shortening the window of opportunity for antigen recognition, thus maximizing CD8+ T-cell sensing for elimination of HIV-1 reactivated cells.

### Inter-individual Divergences in *ex vivo* CD8+ T-Cell Recognition and Killing of LRA-Reactivated Cells

Although the use of CTL supports rapid recognition of HIV-1 reactivated cells based on LRA potency, these CTL are highly functional and sensitive to HIV-1 antigen presentation and may not fully recapitulate the diversity of HIV-1–specific CD8+ T cells in infected individuals.

**Figure 4:**
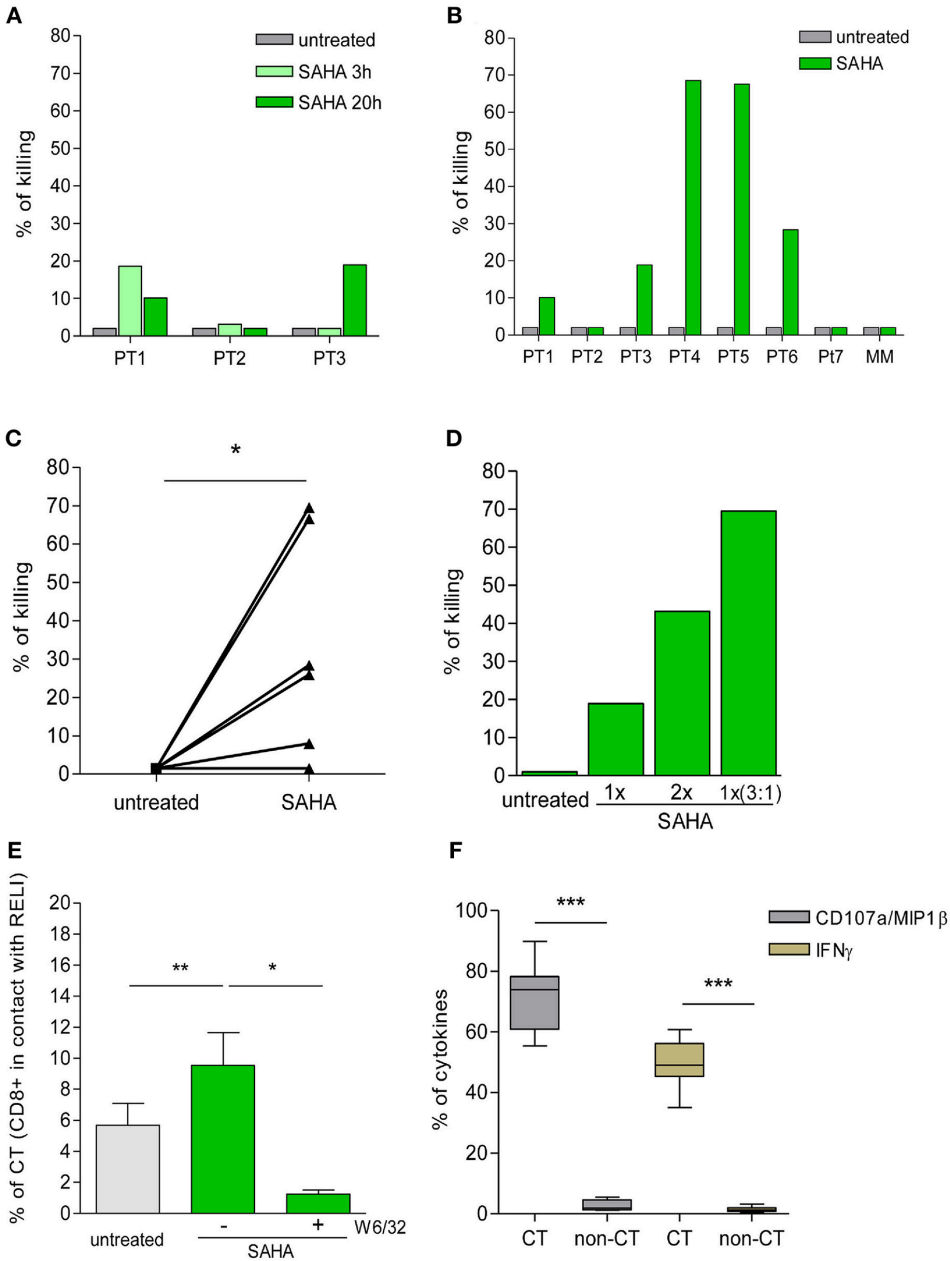
Killing of HIV-1 reactivated cells by *ex vivo* CD8+ T cells. **(A)** Percentage of killing of untreated or SAHA-treated HIV-1 RELI cells at 3 and 20 h by *ex vivo* CD8+ T cells from HIV-1-infected individuals (*n* = 3). **(B)** Percentage of killing of untreated or SAHA-treated HIV-1 RELI cells by *ex vivo* CD8+ T cells at 20 h (*n* = 7). **(C)** Paired comparison of the percentage of killing of untreated or SAHA-treated HIV-1 cells by *ex vivo* CD8+ T cells shown in **(B)**. The graph represents the percentage of the killing of RELI cells. The *p*-values were calculated using the paired *t*-test. **(D)** Percentage of killing of untreated or SAHA-treated RELI cells by *ex vivo* CD8+ T cells from PT3. Conditions with a 1X SAHA and 2X SAHA and co-cultures at an E:T ratio of 3:1 in 1X SAHA conditions are shown. **(E)** Percentage of CD8+ T cells in contact with untreated or SAHA-treated HIV-1 RELI cells in the absence or presence of a W6/32 antibody after 20 h of co-culture. The *p*-values were calculated using the paired *t-*test. **(F)** Percentage of CD107a/MIP1β and IFNγ secretion by CD8+ T cells after 20 h in contact or not (CT/non-CT) with SAHA-reactivated cells. The *p*–values were calculated using the paired *t*-test. Only significant values (^*^p < 0.05, ^**^p < 0.005 and ^***^p < 0.0005) are shown.

To delineate the kinetics of recognition and killing of LRA-reactivated cells by CD8+ T cells, we initially co-cultured SAHA-treated cells with *ex vivo* CD8+ T cells obtained from three of the eight HIV-1-infected individuals tested (participants characteristics in Table 1). As shown in Figure 4A, we observed variation in the kinetics of recognition and killing of reactivated cells by *ex vivo* CD8+ T cells. While we observed killing in response to SAHA at 3 h for PT1, we did not observe elimination of reactivated cells for PT2; in addition, elimination of reactivated cells was only observed after 20 h of co-culture for PT3. These divergences suggest functional differences between CD8+ T cells from HIV-1-infected individual associated a particular profile of recognition and killing of LRA-reactivated cells (41).

**Table 1:** Characteristics of study participants.

**Participant ID**	**Age at sample**	**Gender, Ethnicity**	**ART regimen**	**HLA-B serotype**	**VL (copies/ml)**
PT1	34	M, CAU	TDF/FTC/RAL	B^*^27:05	<40
PT2	43	F, CAU	Naïve	B^*^27:02	<40
PT3	36	M, CAU	ABC/3TC/DTG	B^*^27:05	<40
PT4	38	M, CAU	TDF/FTC/RAL	B^*^27:05	<40
PT5	36	M, CAU	Naïve	B^*^27:05	44000
PT6	40	M, CAU	TDF/FTC/ELV/COB	B^*^27:05	<40
PT7	37	F, CAU	Naïve	B^*^27:05	982
MM	47	M, CAU	AZT/DDC	Non-B^*^27	<40

*VL, viral load; MM, mismatch; TDF, tenofovir; FTC, emtricitabine; RAL, raltegravir; ABC, abacavir; 3TC, lamivudine; DTG; dolutegravir; ELV, elvitegravir; COB, cobicistat; AZT, zidovudine; DDC, zalcitabine*.

Next, we extended our initial analyses to an additional group of HIV-1-infected individuals (Table 1). As in the previous experiments, we found high inter-individual variability in the killing capacity of HIV-1-reactivated cells by CD8+ T cells ranging from 2% (PT2 and PT7) to 70% (PT4 and PT5) (Figure 4B). The killing observed was specific for CD8+ T-cell recognition in the context of HLA-class I presentation, as supported by the lack of killing observed by mismatch CD8+ T cells (MM). Despite this biological divergence, we observed a significant increase in the killing of SAHA-treated cells by CD8+ T cells (*p* < 0.05, Figure 4C). Moreover, we were able to enhance the magnitude of CD8+ T-cell killing *ex vivo*, either by increasing the concentration of SAHA 2-fold or the effector-to-target ratio 3 times (Figure 4D). These results demonstrate the possibility of modulating and improving the efficacy of CD8+ T-cell killing in response to HIV-1 inducible expression by higher LRA dosage or higher frequency of effector cells. We also carried out a detailed study of cell-to-cell interactions in our co-culture system. We observed an increase in the number of CD8+ T-cell contacts with target cells upon reactivation of SAHA (*p* < 0.005). These contacts were specifically blocked by the addition of an anti-HLA-A/B/C antibody (*p* < 0.05) (Figure 4E), thus demonstrating the dependency of cell-to-cell contact on the interactions between TCR and HLA-class I/peptide complexes after LRA treatment. In addition, CD8+ T cells that established contact were highly functional, as measured by the levels of CD107a/MIP1β and IFNγ when compared with CD8+ T cells lacking contacts with target cells (*p* < 0.0001) (Figure 4F).

These findings support an overall increase in the killing of HIV-1-infected cells by CD8+ T cells upon LRA induction of viral reactivation despite inter-individual divergences. Moreover, our data point to the opportunity to optimize the immune clearance of HIV-1 reactivated target cells by CD8+ T cells by increasing the LRA dosage and the frequency of effector CD8+ T cells.

### Expression Profiles of Inhibitory Receptors in HIV-1–Specific CD8+ T Cells Modulates the Killing of HIV-1 Reactivated Cells

Inter-individual differences in functional ability to recognize and eliminate HIV-1-infected cells *ex vivo* by CD8+ T cells could be the result of differences in time to initiation of ART (42), in the persistence of antigen exposure and in the chronic pro-inflammatory environment despite ART (30). The pro-inflammatory environment and the long-term exposure to antigen lead to dysfunctional HIV-1–specific CD8+ T-cell responses and eventually to immune exhaustion.

**Figure 5:**
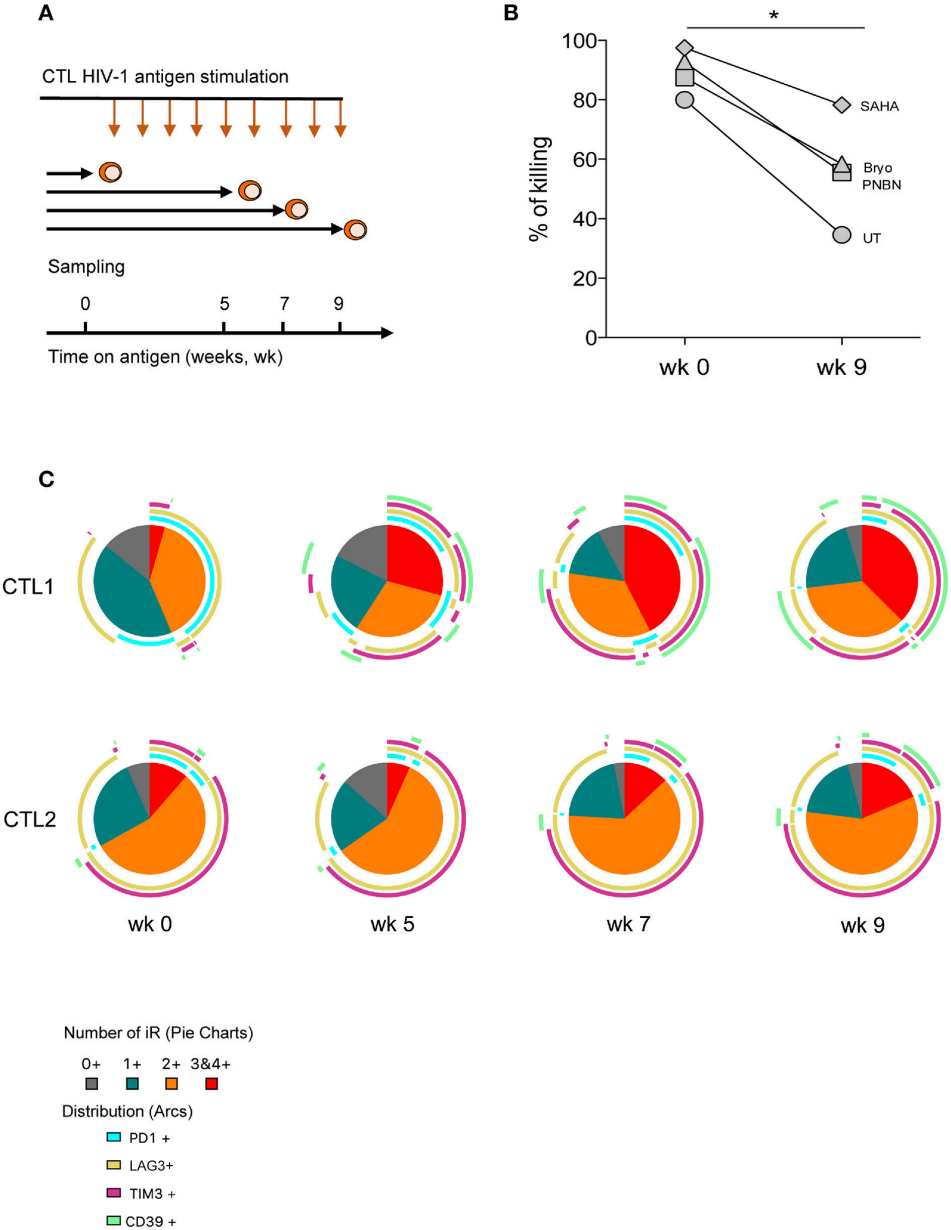
CD8+ T-cell dysfunction impairs clearance of HIV-1 reactivated cells associated with an increase in the co-expression of inhibitory receptors. **(A)** Schematic representation of the *in vitro* exhaustion process, where CTL were exposed to HIV-1 cognate peptide over a period of 9 weeks. **(B)** Percentage of killing of HIV-1 reactivated cells by CTL after antigen stimulation at week 0 and at 9 weeks. The graph includes one representative experiment. The *p*-value was calculated using the paired *t*-test. Statistical significance is shown in the figure (^*^p < 0.05). **(C)** Pie charts representing the fraction of cells expressing different combinations of PD-1, LAG3, TIM-3, and CD39 at weeks 0, 5, 7, and 9 after antigen stimulation.

To delineate the determinants of CD8+ T-cell dysfunction in response to LRA-reactivated cells, we cultured CTLs with HIV-1 cognate peptide for 9 weeks with weekly rounds of peptide re-stimulation to simulate *in vitro* long-term antigen exposure (Figure 5A). At weeks 0, 5, 7, and 9 we performed an immune phenotype of inhibitory receptors (PD-1, LAG3, and TIM-3) and the immune-metabolic marker CD39. All these markers have been previously associated with the exhaustion of HIV-1–specific CD8+ T-cell responses (28, 43). As shown in Figure 5B, we found a significant reduction in the killing of LRA-reactivated cells by CTL after 9 weeks of continuous antigen exposure (*p* < 0.005). Despite the overall reduction in the killing by CTL after 9 weeks, we did observe an increase in killing between untreated and SAHA conditions (35–58%) (Figure 5B).

We also monitored phenotypic changes in CTL and found a contraction in the frequency of subpopulations without expression or expressing a single inhibitory receptor concomitant with the expansion of cells co-expressing three or four inhibitory receptors over time (Figure 5C, pie charts). This phenotype of expansion/contraction was coincident between CTL1 and CTL2, which vary in HIV-1 antigen specificity. However, the specific combinations of inhibitory receptors diverge between CTL lines and time, thus highlighting the diversity of T-cell inhibitory pathways. Also, CD39+ subpopulations appeared after 5 weeks in cells already expressing two or more inhibitory receptors (Figure 5C, arcs). These data suggest the presence of terminally exhausted CD8+ T cells identified by CD39 expression, as previously described (43).

**Figure 6:**
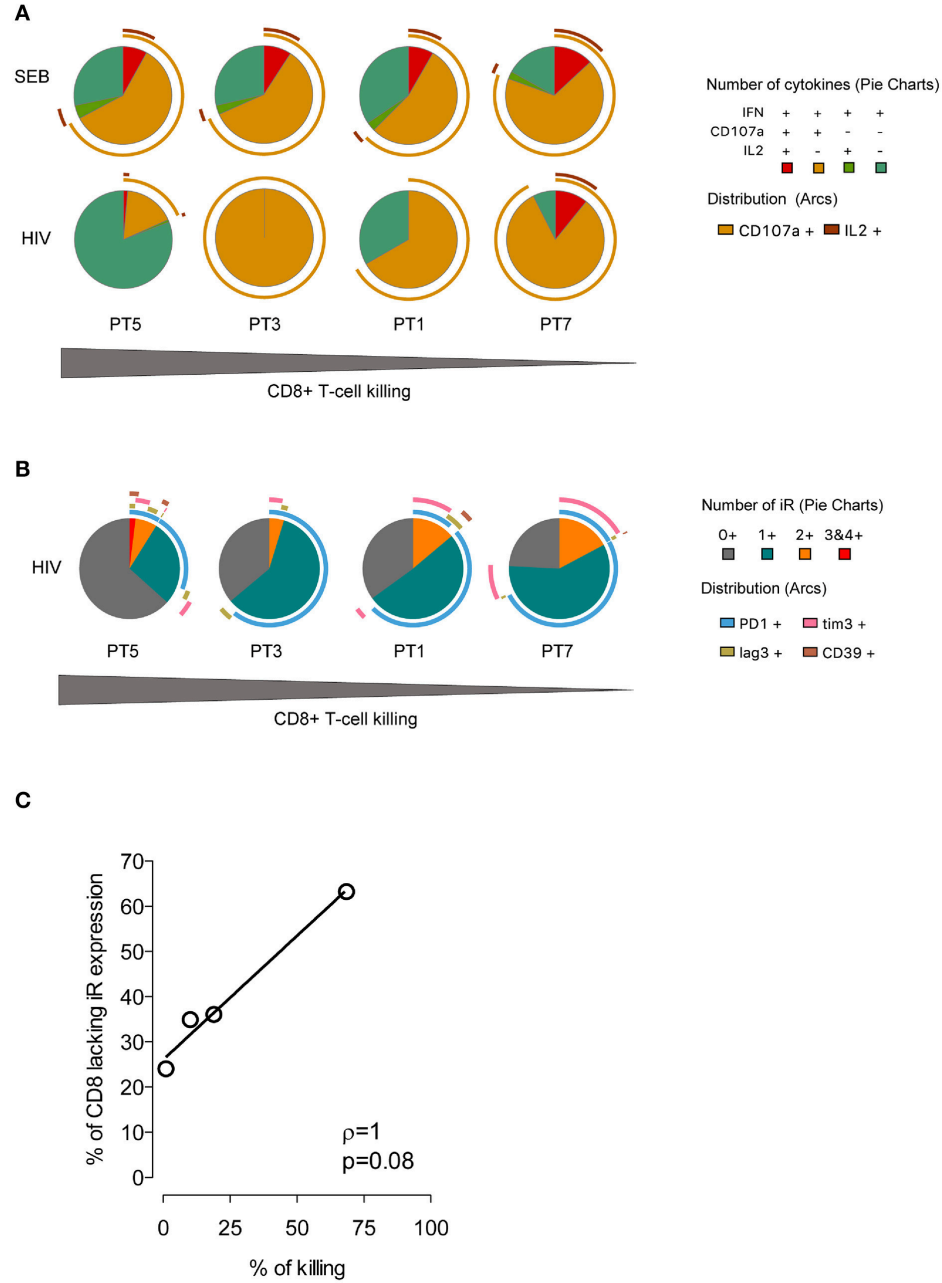
Expression profiles of cytokines and inhibitory receptors in HIV-1–specific CD8+ T cells. **(A)** Functional profiles of HIV-1–specific CD8+ T cells. Pie charts represent functional profiles associated with the secretion of IFNγ, CD107a, and IL-2 in relation with IFNγ+ single, double (IFNγ+CD107a+IL-2-,IFNγ+CD107a- IL-2+), and triple positive cells (IFNγ+CD107a+IL−2+). Data from four HIV-1-infected individuals following 6 h of stimulation with SEB or a pool of HIV-1 Gag peptides is shown. **(B)** Profiles of inhibitory receptor (iR) expression in HIV-1–specific CD8+ T cells from infected individuals taken from IFNγ positive cells. For **(A,B)** the pie slices represent the frequency of cells with expression of one or more markers, and the pie arcs represent the specific markers expressed. The gray gradient below indicates the magnitude of killing of HIV-1 reactivated cells by four HIV-1-infected individuals. **(C)** Correlation of the frequency of HIV-1–specific CD8+T cells lacking iR expression and the magnitude of SAHA-reactivated cell killing. The line indicates the fit of the data to a linear regression. The Spearman correlation coefficient (r) and the two-tailed *p*-value are indicated.

Based on these data and the biological diversity found in the recognition and killing of LRA-reactivated cells by *ex vivo* CD8+ T cells, we measured HIV-1–specific CD8+ T-cell function in response to a pool of Gag overlapping peptides. We monitored expression of CD107a, IFNγ, and MIP1β cytokines and of PD-1, LAG3, TIM-3, and CD39 by multiparametric flow cytometry. As shown in Figure 6A, we observed variation in the profile of cytokine production among individuals in response to HIV-1. Meanwhile, the functional response to SEB control stimuli was very homogeneous, as expected. Furthermore, we did not find a correlation between the frequency of polyfunctional cells among the HIV-1–specific CD8+ T cells and the killing in response to LRA-reactivated cells, as it has previously been associated with spontaneous control of HIV-1 replication (44, 45). Importantly, individuals harboring HIV-1–specific CD8+ T cells lacking expression of inhibitory receptors retained the highest suppressive capacity against HIV-1 LRA-reactivated cells (Figure 6B). Thus, PT5 responded to LRA treatment with a 68% *in vitro* suppressive capacity and 63% of HIV-specific CD8+ T cells lacking inhibitory receptors. Participants PT3, PT1, and PT7 with 36, 35, and 24% of HIV-1–specific CD8+ T cells lacking inhibitory receptors had suppressive capacities achieving 19, 10, and 2% of killing of SAHA reactivated cells. Although limited by sample size, our data delineate a correlation between the percentages of HIV-1–specific CD8+ T cells lacking inhibitory receptors and their immune responsiveness to killing LRA treatment (*p* < 0.005, Figure 6C).

According to these data, changes in the profile of inhibitory receptors in HIV-1-specific CTL and primary CD8+ T cells modulate their antiviral potency. Therefore, the levels of inhibitory receptor co-expression in CD8+ T cells can limit the magnitude of the killing of HIV-1-reactivated cells.

## Discussion

Clinical use of LRA revealed detectable perturbations in the HIV-1 reservoir with no measurable impact on reservoir size (5–8). Therefore, the potential of shock and kill strategies to purge HIV-1 reservoirs should be carefully evaluated to delineate current therapeutic limitations. On the one hand, LRA should be able to induce HIV-1 latency reversal for recognition by immune effector cells. On the other, the functional characteristics of CD8+ T cells should ensure the effective clearance of reactivated cells. Here, we report the barriers that account for the immune recognition of HIV-1 latently infected by CD8+ T cells in the context of shock and kill cure strategies. Our data demonstrate the potency of LRA for speeding up the recognition of reactivated cells and CD8+ T-cell dysfunction associated with the expression of inhibitory receptors as limitations to ensure the killing of HIV-1 reactivated cells. Thus, we characterize the level of HIV-1 protein expression upon treatment with LRA as a mechanism that is essential for rapid and potent killing of reactivated cells by CD8+ T cells. Moreover, our findings suggest high inter-individual variation in CD8+ T-cell responsiveness to HIV-1 reactivation, where the expression of inhibitory receptors modulates the magnitude of killing.

As shown by previous reports, we found how reactivation of latently infected cells by LRA enables HIV-1 antigen presentation for recognition and killing by CD8+ T cells (7, 46). Our data are consistent with those from previous studies and suggest that once LRA induced HIV-1 antigen production, there was no interference in antigen presentation in the context of HLA-class I molecules for recognition by CD8+ T cells (22). In addition, we were able to characterize in detail the characteristics of the so-called window of opportunity between reactivation and killing for CD8+ T cells. In our experimental framework, the killing of reactivated cells can happen as early as 3 h after co-culture, under conditions where LRA treatment achieves the highest reactivation level. The correlation between LRA potency measure as HIV-1-fold induction, and the speed of recognition by CD8+ T cells uncover the presence of a threshold of antigen production after administration of LRA for killing. Although our experimental data propose a threshold above a 2-fold induction of HIV-1 reactivation for CD8+ T-cell killing, we need to remember that the data were obtained in the context of two particular CTL. Our results constitute a proof of principle on the need for potent LRAs to ensure the rapid recognition of HIV-1 reactivated cells for effective shock and kill strategies, even in the context of fully functional CD8+ T-cell responses. However, we cannot extrapolate the current findings as this theoretical threshold may vary across HIV-1-infected individuals with differences in TCR affinities (31, 47). It is necessary to perform further experiments that evaluate diversity in TCR affinities between CD8+ T-cell clonotypes in HIV-1-infected individuals in the context of LRA in order to ensure accurate delineation of the early reactivation events and broad immune recognition of LRA-reactivated cells. The consistency of our data across the CTLs used may indicate that clearance of reactivated cells is independent of CTL antigen specificity when an adequate threshold of reactivation is reached.

Furthermore, we did not find differences in the levels of cytokines secreted by CTL between untreated and HDACi-treated conditions, which may constrain their use as biosensors of HIV-1 reactivation events, as previously suggested (40). As mentioned, the use of highly sensitive CTL in our experiments may limit their capacity to detect small differences in the number of antigens presented between the conditions tested. Thus, we propose to explore additional markers of CD8+ T-cell activation to validate their use as biosensors of HIV-1 reactivation. We also detected a significant increase in cytokine secretion and killing by CTL under Bryo conditions in the absence of HIV-1 reactivation (22, 24). Such a pre-activation status driven by residual Bryo despite extensive washout, or by the direct effect of Bryo on target cells did not show a measurable benefit concerning faster or better killing when compared with HDACi alone. These data alert to the potential effect of using Bryo, which leads to undesirable and non-specific CD8+ T-cell activation and cytokine release, as suggested in other studies (23, 24).

Together with LRA potency, CD8+ T-cell functional status is essential to guaranty the efficacy of shock and kill strategies to clear the HIV-1 reservoir. Our data indicate biological diversity across *ex vivo* CD8+ T cells from HIV-1-infected individuals in response to LRA-reactivated cells. In our experimental framework, we can exclude some of the barriers for CD8+ T-cell function, including viral escape (20), anatomical compartmentalization (48), and lack of viral protein translation (21). Therefore, the differences observed here are mainly due to functional differences between CD8+ T cells from HIV-1-infected individuals. Although we demonstrate an increase in the killing of reactivated cells by *ex vivo* CD8+ T cells after treatment with SAHA, we found differences in the kinetics and the total magnitude of killing between individuals. These differences in response to LRA treatment reflect the heterogeneity of the HIV-1–specific CD8+ T cell responses, even in the context of the HLA-B^*^27 allele expressed in our target cells, which has been associated with strong antiviral responses and elite control of HIV-1 (14).

Multiple factors account for the functional diversity of HIV-1–specific-CD8+ T-cell responses irrespective of the clinical stage of HIV-1, including the low frequency of HIV-1–specific CD8+ T cells upon antigen clearance by long-term ART and continuous immune CD8+ T-cell activation despite ART (18, 19). Moreover, HIV-1–specific CD8+ T-cell dysfunction is associated with a high level of expression of inhibitory receptors including PD-1, TIM-3, LAG-3, and TIGIT (26, 27, 29). These markers have been associated with CD8+ T-cell dysfunction and terminal exhaustion in chronic infections (25, 30). Our *in vitro* data demonstrate that upon continuous exposure to HIV-1 antigens, there is a contraction of subpopulations lacking inhibitory receptors. This is concomitant with the expansion of CTL subpopulations co-expressing at least three inhibitory receptors, including PD-1, LAG3, TIM-3, and the metabolic marker CD39 (43). In our experimental setting, the expansion of CTL co-expressing inhibitory receptors over time diminished the magnitude of the killing of LRA-reactivated cells. However, our *in vitro* model does not account for the pro-inflammatory environment of HIV-1 infection. Further studies including the use of pro-inflammatory cytokines and characterization of the expression of inhibitory receptors in tetramer-sorted HIV-1–specific cells expose to HIV-1 peptides will help to clarify whether our findings can be extended to HIV-1–specific circulating CD8+ T cells.

Despite the limitations mentioned, our findings were consistent with the direct use of *ex vivo* CD8+ T cells, where the frequency of HIV-1–specific CD8+ T cells lacking inhibitory receptors directly correlated with the magnitude of killing of LRA-reactivated cells. This is the first study to our knowledge that suggest an inter-dependency between CD8+ T-cell function and the expression patterns of inhibitory receptors in response to HIV-1 reactivation (28).

The identification of novel inhibitory receptors as markers of HIV-1–specific CD8+ T-cell dysfunction has increased in recent years. The interest has risen due the unprecedented success of clinical trials targeting inhibitory receptors in cancer regression (49, 50). Consequently, additional efforts are needed to clarify the impact of inhibitory receptor blockade in HIV-1-infected individuals. Immunotherapy based on blockage of inhibitory receptors has proven effective in various types of cancer. The use of PD-1 inhibitors have demonstrated to be safe and effective in HIV-infected individuals with lung cancer (51). Moreover, the use of an anti-PD-L1 in HIV-1-infected individuals has already been evaluated in a Phase I clinical trial (52), in which anti-PD-L1 had a limited effect on the recovery of HIV-1–specific CD8+ T-cell responses (2 out of 6 patients). Consequently, the delineation of the functional characteristics of HIV-1–specific CD8+ T cells behind successful shock and kill strategies will play a key role in the assessment of treatment efficacy. The efforts to define these traits should accompany the development of ultrasensitive technologies for the monitoring of small perturbations in the size of the HIV-1 reservoir in response to low fractions of reactivated cells killed by CD8+ T cells in blood or tissues. These technologies will be essential to guide the outcome of curative strategies.

In summary, our study suggests the need for a strong flush of latent virus to speed up recognition of HIV-1 reactivated cells and an adequate functional capacity of HIV-1–specific CD8+ T cells to maximize elimination of LRA-reactivated cells. An adequate trade-off between shock and kill is essential to ensure the success of potential curative strategies. The immune characterization of HIV-1–specific CD8+ T-cell responses, including patterns of inhibitory receptors associated with the killing of reactivated cells, will allow the design of novel immune-monitoring and immuno based therapeutics to ensure effective antiviral responses before latency reversal in HIV-1-infected individuals.

## Materials and Methods

### Virus and Cell Lines

The virus NL43_GFP_ was generated by co-transfection of p83–2 and p83–10_eGFP_ plasmids in MT4 cells (NIH AIDS Reagent program, USA) based on electroporation, as previously described (38, 39). The cell-free supernatant was concentrated by spinoculation at 24,000 rpm at 4°C for 90 min, and stored at −80°C. The 50% tissue culture infective dose (TCID_50_) was determined on TZM-bl cells (NIH AIDS Reagent program, USA) as described in Kloverpris et al. (31). The HIV-1-permissive U937 cell line transfected with the HLA-B^*^27:05 gene (kindly provided by Paul Bowness, UK) (53) was cultured in RPMI media supplemented with 10% fetal calf serum (FCS) (HyClone), 2 mM L-glutamine, 100 U/ml penicillin, 100 μg/ml streptomycin, and 0.5 mg/ml geneticin (Invitrogen, Life Technologies). The HIV-1 cytotoxic CD8+ T-cell lines (CTL) used were restricted by HLA-B^*^27:05. The CTL1 was specific for the KK10 epitope in Gag and CTL2 for the KY9 epitope in Pol. In addition, CTL3 was restricted by HLA-B^*^57:01 and specific for the KF11 epitope in Gag (31, 53).

### The HIV-1 Shock and Kill RELI Model

The U937 HLA-B^*^27:05 permissive cell line was infected with NL43_GFP_ by magnetofection at a dose equivalent to a nominal multiplicity of infection (MOI) of 0.1, as previously reported (31, 32). After magnetofection, cells were washed twice with 1X PBS and cultured in R10 + geneticin (0.5 mg/ml). At day three post-infection, the GFP-negative population, which included the non-productively infected cells enriched in HIV-1 integrated provirus, was sorted with a FACS Aria II Cell Sorter (BD Biosciences) and cultured in the presence of 1 μM of RTV (Sigma, Spain). After 4 days, we obtained a culture enriched in “resting-like” cells (RELI). The mean levels of cell-associated total HIV-1 DNA in RELI-lysates was 5.1–log ± 0.19 log_10_ copies/10^6^ RELI as measured by droplet digital PCR previously described in Martínez-Bonet et al. (54). RELI cells were treated with LRA in the presence of RAL at 100 nM (Sigma, Spain) to monitor HIV-1 reactivation or “shock.” The LRA panel included the HDACi: panobinostat (PNBN) at 30 nM (SelleckChem, USA), trichostatin A (Tricho) at 250 nM (Sigma, Spain), SAHA (suberoyl aniline hydroxamic acid) at 1 μM (SelleckChem, Spain), and romidepsin (RMD) at 40 nM (SelleckChem, Spain), and the PKC agonist bryostatin A (Bryo) at 10 nM (Sigma, Spain). RELI cells were treated for 48 h with LRA except for RMD-treated cultures, where the drug was washed-off after 4 h to avoid cellular toxicity.

To monitor the “shock” or HIV-1-fold induction at 48 h after treatment with LRA, the RELI cells were harvested and stained with a Live/Dead probe (APC-Cy7, Invitrogen). After washing, cells were fixed and permeabilized with the Fix&Perm kit (A and B solutions, Thermo Fisher Scientific) for intracellular HIV-1 p24 (PE, clone KC57-RD1, Beckmann Coulter). Cells were acquired on an LSR II flow cytometer (Beckmann Coulter), and data were analyzed using FlowJoV (Tree Star Inc.). The HIV-1-fold induction was calculated based on the following equation: % of p24+ or GFP+ in CD4+ under LRA conditions/% of p24+ or GFP+ under untreated conditions. To proceed with the co-cultures to monitor the “killing” of reactivated cells by CD8+ T cells the RELI cells were extensively washed with 1X PBS and left in R10 media plus RAL. Thus, we cultured LRA-reactivated RELI cells in the absence or presence of HIV-1-specific CTLs or B^*^27-HLA-matched *ex vivo* CD8+ T cells obtained from HIV-1-infected individuals. Cells were co-cultured to a 1:1 effector: target ratio for 20 h. After 20 h of co-culture, we simultaneously measured by flow cytometry the “shock” by intracellular p24, and the “kill” by the reduction of p24-positive RELI cells in the absence or presence of effector cells. We also assessed LRA-related toxicity both in uninfected U937 cells and in the RELI model by measuring cellular viability using flow cytometry and staining with a Live/Dead probe (APC-Cy7, Invitrogen) at the end of the co-culture with RELI and CTL.

### CD8+ T-Cell Killing and Activation

We performed CD8+ T-cell killing experiments of LRA-reactivated cells with CTL or *ex vivo* CD8+ T cells at 20 h after culture. We also monitored the kinetics of CD8+ T-cell killing of LRA-reactivated cells with CTL or *ex vivo* CD8+ T cells after 3, 6, and 20 h after co-culture. We monitored CTL and CD8+ T-cell activation by intracellular expression of CD107a, MIP-1β, and IFNγ. For analysis of HLA-class I restriction, the W6/32 antibody (BioLegend, Spain) or the isotype control LEAF purified IgG2aÎ (BioLegend) was added to the co-culture at 10 μg/ml. For analysis of CD8+ T-cells in contact with RELI cells, we analyzed the percentage of doublets gated cells that expressed the CD8+ surface marker in the target cells gate. For analysis of early CD107a secretion, the antibody CD107a (PerCP-Cy5.5, BD Biosciences) was added to the co-culture. For the immune-phenotype, samples were collected and washed twice with 1X PBS and incubated for 3 h in the presence of the protein transport inhibitor Golgiplug (Brefeldin A solution, BD Biosciences, Spain) and Golgistop (Monensin solution, BD Biosciences, Spain). Cells were then harvested, stained with a Live/Dead probe (APC-Cy7, Invitrogen). Next, cells were washed and stained to identify the surface marker CD8 (Pacific blue, clone RPA-T8, BD Biosciences). After washing, cells were fixed and permeabilized with the Fix&Perm kit (A and B solutions, Thermo Fisher Scientific) for intracellular cytokine staining of MIP1β (FITC, clone 24,006, R&D Systems), IFNγ (PE-Cy7, clone 4S.B3, BD Biosciences), and HIV-1 p24 (PE, clone KC57-RD1, Beckmann Coulter). Cells were acquired on an LSR II flow cytometer (Beckmann Coulter). Data were analyzed with FlowJoV (Tree Star Inc.). The killing of reactivated HIV-1 cells by CTL/CD8+ was calculated based on the following equation: 100—[(% p24+ in CD4+ co-cultured with CD8+/% p24+ in CD4+ in the absence of CD8+) × 100].

### *In vitro* CTL Exhaustion Experiments

To characterize potential markers associated with CTL dysfunction in response to LRA-reactivated cells, we exposed CTL1 and CTL2 to cognate HIV-1 antigen over nine weeks. To do so, we stimulated CTL with a 1:1 mixture of irradiated autologous B-cell lines (BCLs) pulsed with cognate HIV peptide (10 μg/ml) and irradiated PBMCs from three healthy HIV-seronegative donors weekly. We used flow cytometry to evaluate variations in the expression of the inhibitory receptors PD-1, TIM-3, LAG-3, and the immune-metabolic marker CD39. Briefly, cells were taken from the culture at weeks 0, 5, 7, and 9 and stained with a Live/Dead probe (APC-Cy7, Invitrogen) and surface markers CD3 (Alexa700, BD Biosciences), CD4 (APC-Cy7, BD Biosciences), CD8 (V500, BD Biosciences), PD-1 (BV421, BD Biosciences), TIM-3 (Alexa 647, BD Biosciences), LAG-3 (PE, BD Biosciences), and CD39 (FITC, BD Biosciences) and incubated at room temperature for 25 min. Samples were washed twice with 1X PBS, fixed in 1% formaldehyde, and acquired on an LSR Fortessa. Data were analyzed with FlowJoV (Tree Star Inc.). Patterns of co-expression of PD-1, LAG-3, TIM-3, and CD39 were analyzed using Pestle and SPICE v5 software (55).

### Immunophenotype of HIV-1–Specific CD8+ T-Cell Responses

The PBMCs from HIV-1-infected individuals were stimulated with HIV-1 Gag peptide pool (2 μg/peptide/ml, EzBiolab), or the positive control SEB (enterotoxin B from *Staphylococcus aureus*, Sigma, Ref. S4881) or no stimuli in the presence of CD28/49d co-stimulatory molecules (1 μl/ml, BD Biosciences), Golgistop solution (Monensin A, BD Biosciences, Spain), and anti-human CD107a (PE-Cy5, BD Biosciences) for 6 h at 37°C in a 5% CO_2_ incubator and overnight at 4°C. Stimulation cells were then washed with 1X PBS and stained for 25 min with the Live/Dead probe (APC-Cy7, Invitrogen). Next, cells were washed with 1X PBS and surface stained for 25 min with CD3 (A700, BD Biosciences), CD4 (APC-Cy7, BD Biosciences), CD8 (V500, BD Biosciences), PD-1 (BV421, BD Biosciences), LAG3 (PE, BD Biosciences), TIM-3 (A647, BD Biosciences), and CD39 (FITC, BD Biosciences). Subsequently, cells were washed twice in 1X PBS and fixed and permeabilized with the Fix&Perm kit (A and B solutions, Thermo Fisher Scientific) for intracellular cytokine staining of IFNγ (BV711, BD Biosciences) and IL-2 (BV650, BD Biosciences). After 20 min at room temperature in the dark, stained samples were washed twice with 1X PBS, resuspended in 1% formaldehyde and acquired on an LSR Fortessa. Data were analyzed with FlowJoV (Tree Star Inc.). Patterns of simultaneous expression of IFNγ, CD107a, and IL-2, or combinations of PD-1, LAG-3, TIM-3, and CD39 were analyzed using Pestle and SPICE v5 software (55).

### Statistics

Statistical analyses were performed using Prism v4 (GraphPad Software, Inc.). Reported *p*-values were calculated using the Wilcoxon single-rank test in paired comparisons and the *t-*test and Mann-Whitney test for independent median comparisons. Also we used one-way ordinary analysis of variants (ANOVA), and one-way ANOVA for multiple group comparisons adjusting the pairwise analyses by Tukey. The relationship between HIV-1 induction measured according to the expression of p24 and GFP, and the relationship between the lack of inhibitory receptors in CD8+ and percentage of killing were assessed using the Spearman correlation coefficient. All statistical tests were under a significance level of 0.05.

## Ethics Statement

All methods and experimental protocols of the study were approved by the Ethics Committee of Hospital Germans Trias i Pujol. For the study, both infected and healthy subjects provided their written informed consent to participate. The study was conducted according to the principles expressed in the Declaration of Helsinki.

## Author Contributions

AR and JGP: design of the study. AR, OB-L, EJ-M, RP, and CG: performance of the experiments. AR, OB-L, EJ-M, JGP, MG, BM, DO, CG, and RB: analysis of the data and results. AR, OB-L, BM, JM-P, PG, MG, RB, BH, BC, and JGP: manuscript drafting and discussion.

### Conflict of Interest Statement

RB and BH were employed by the company Merck. JGP and JM-P report research funding from Merck. JGP and AR have a patent on the methods for screening of HIV-1 latency reversing agents issue (62590081). The remaining authors declare that the research was conducted in the absence of any commercial or financial relationships that could be construed as a potential conflict of interest.
