# Supplementary material for: Antigen Production After Latency Reversal and Expression of Inhibitory Receptors in CD8+ T Cells Limit the Killing of HIV-1 Reactivated Cells
Source: Front Immunol. 2019 Jan 22;9:3162. doi: 10.3389/fimmu.2018.03162 (PMC6349966; doi:10.3389/fimmu.2018.03162)

**SUPPLEMENTAL DATA**

**
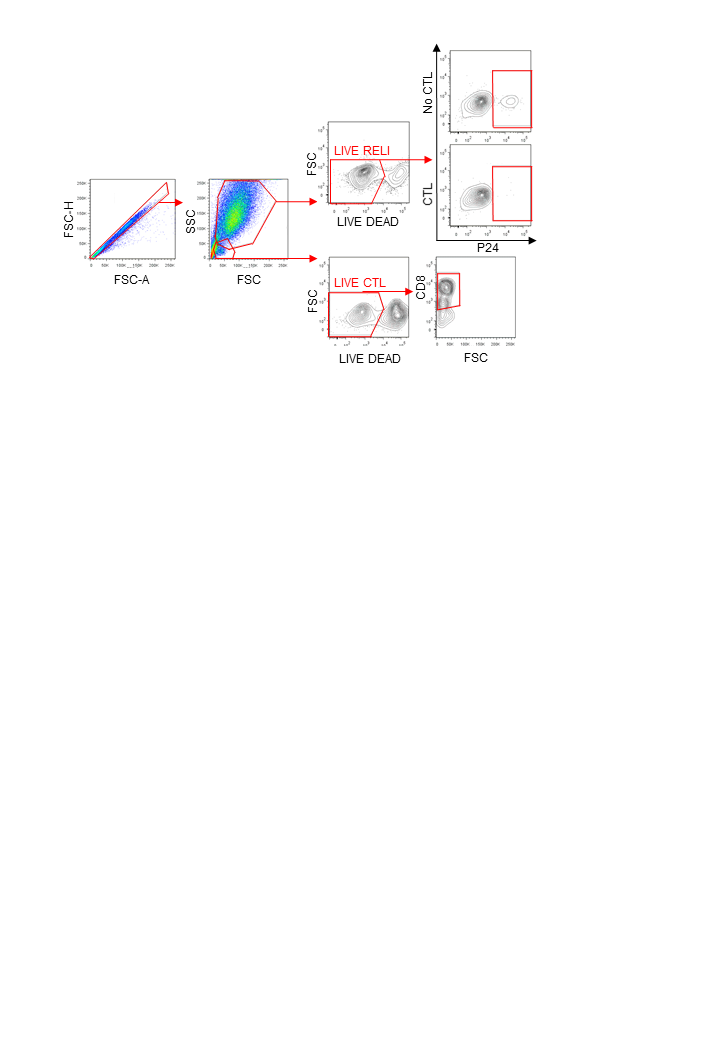
**

**S1 Figure. Gating strategy in the “HIV-shock and CTL kill” RELI model.** The “shock” in target-RELI cells was measured by intracellular p24 and GFP expression levels by flow cytometry. The “kill” was evaluated with the reduction of HIV-p24 positive cells in the absence or presence of effector-CTL cells. Cellular viability was monitored both in target and effector cells.


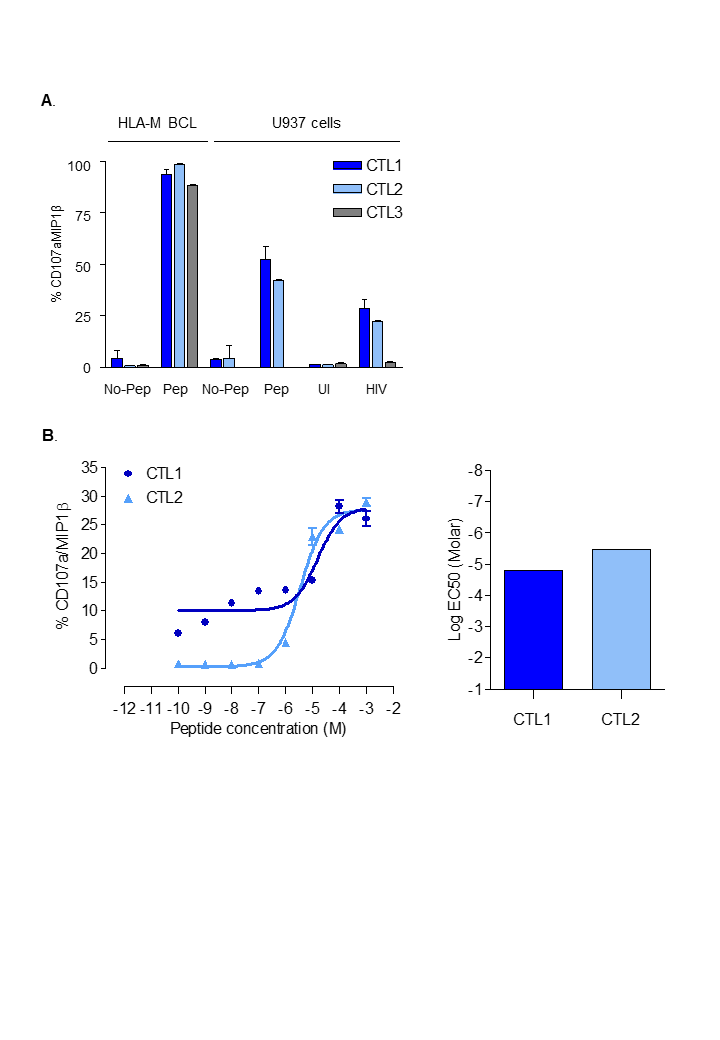


**S2 Figure. CTL cytokine secretion profiles. (A)** CD107a/MIP1β secretion from CTL1, CTL2 and CTL3 after a 3h co-culture with different targets cells: peptide-pulsed HLA-matched B-cell lines (HLA-M BCL), and the HLA-B27 U937 cell line, peptide-pulsed or not, and HIV-1 infected or not. CTL1 and CTL2 matched with HLA-B27 U937 cells, while CTL3 is a mismatch for this cell line. **(B)** Curves of CD107a/MIP1β secretion from CTL1 and CTL2 after co-culture with U937 cells pulsed with serially diluted cognate peptide. U937 cells were pulsed with the indicated Log10 serial peptide dilutions for 1h at 37 C before co-culture with CTL1 or CTL2 for 3h. The CD107a/MIP1β secretion profile was determined by flow cytometry. Log EC50 values were calculated and plotted for CTL1 and CTL2.

**S1 Table. Cellular toxicity associated with LRA treatment**


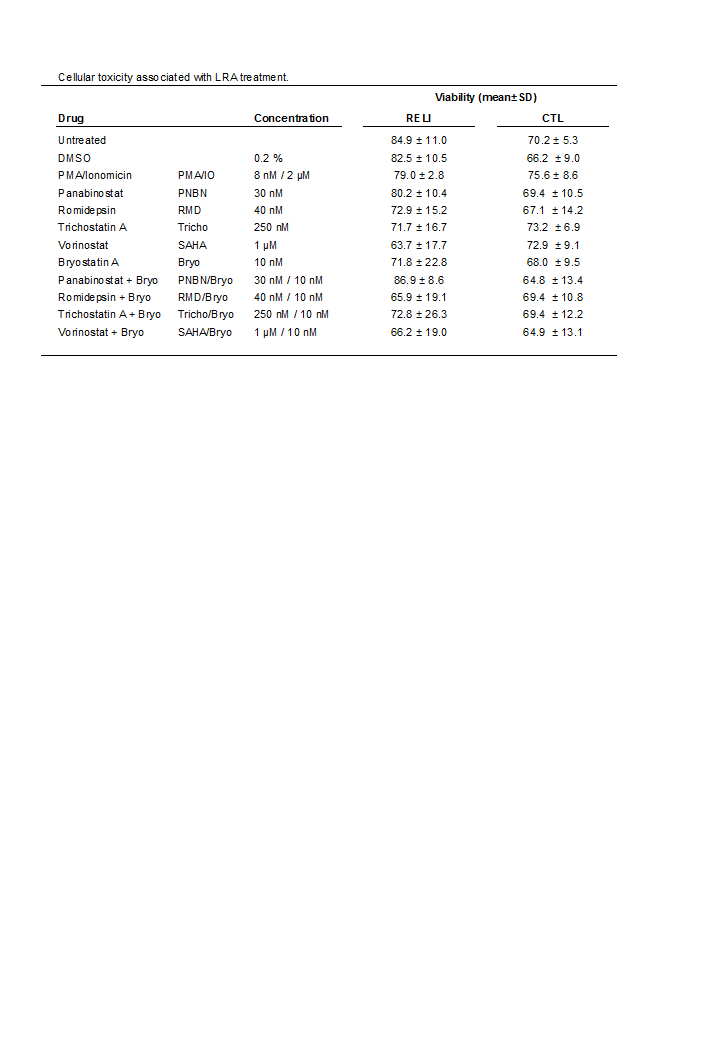

Supplement: Supplementary file 1 [file Data_Sheet_1.doc]
